# Implications of the heterogeneity between guideline recommendations for the use of low dose aspirin in primary prevention of cardiovascular disease

**DOI:** 10.1016/j.ajpc.2022.100363

**Published:** 2022-06-06

**Authors:** Xiao-Ying Li, Li Li, Sang-Hoon Na, Francesca Santilli, Zhongwei Shi, Michael Blaha

**Affiliations:** a Department of Geriatric Cardiology, Chinese PLA General Hospital, Beijing, China; b Medical Affairs & Pharmacovigilance, Pharmaceuticals, Bayer AG, Berlin, Germany; c Seoul National University Hospital, Seoul, Republic of Korea; d Department of Medicine and Aging and Center for Advanced Studies and Technology, D'Annunzio University of Chieti–Pescara, Chieti, Italy; e Department of Cardiology, Ruijin Hospital, Shanghai Jiao Tong University School of Medicine, Shanghai, China; f Johns Hopkins Ciccarone Center for the Prevention of Heart Disease, Johns Hopkins University Medical Center, Baltimore, MD, United States

**Keywords:** Cardiovascular disease, Primary prevention guidelines, Low dose aspirin, Antiplatelet

## Abstract

The most recent primary cardiovascular disease (CVD) prevention clinical guidelines used in Europe, Italy, the USA, China, and South Korea differ in aspects of their approach to CVD risk assessment and reduction. Low dose aspirin use is recommended in certain high-risk patients by most but not all the countries. Assessment of traditional risk factors and which prediction models are commonly used differ between countries. The assessments and tools may not, however, identify all patients at high risk but without manifest CVD. The use of coronary artery calcium (CAC) score to guide decisions regarding primary prevention aspirin therapy is recommended only by the US primary prevention guidelines and the 2021 European Society of Cardiology guidelines. A more consistent and comprehensive global approach to CVD risk estimation in individual patients could help to personalize primary CVD prevention. Wider detection of subclinical atherosclerosis, together with structured assessment and effective mitigation of bleeding risk, may appropriately target patients likely to gain net benefit from low dose aspirin therapy.

## 1 Introduction

Aspirin remains the most widely used and least expensive antiplatelet agent. Low dose aspirin has a unique mechanism of action, consisting of irreversible inhibition of cyclooxygenase-1 (COX-1) enzymes that are expressed in platelets and megakaryocytes, and suppression of prostaglandin and thromboxane-A_2_ production. Since anucleate platelets cannot generate new COX, the inhibitory effect lasts the lifespan of the platelet [1]. Inhibition of megakaryocyte COX-1 allows the long-lasting effect of aspirin throughout the 24-hour dosing interval, despite the short, 20-minute half-life of the drug [2]. The action of aspirin complements the action of other cardiovascular (CV) protective agents, (e.g., statins, antihypertensives, and other antithrombotic treatments), to provide additive reduction of CV risk [3]. In contrast to the inclusion of low dose aspirin as a key component of treatment for secondary prevention of CV events in international guidelines, its role in primary prevention has long been a subject of debate. The findings of 3 primary prevention trials in 2018 [4], [5], [6], [7], [8], which reported neutral net benefit results or evidence of harm for aspirin in patients with no overt cardiovascular disease (CVD), have added to that debate and are reflected in the changes in, and discordance between, major primary prevention guidelines over time and by different organizations.

Estimating the absolute CV risk of the patient is acknowledged as a critical step in the current approach to primary prevention of atherosclerotic CVD (ASCVD) [9]. Numerous primary prevention management guidelines provide recommendations or guidance for treatment, based on ASCVD risk assessment and stratification using a population-based risk assessment system or score. However, with an increased understanding of, and ability to detect, subclinical atherosclerosis, it has become apparent that some patients with extensive subclinical disease have a level of CV risk that is similar to or higher than some patients who have had a CV event [10,11]. In patients with a history of CVD, low dose aspirin for secondary prevention has a well-established and near-universal positive benefit:risk ratio. It may therefore be that low dose aspirin retains a role in primary prevention in a group of patients with extensive subclinical disease.

**Fig. 1:**
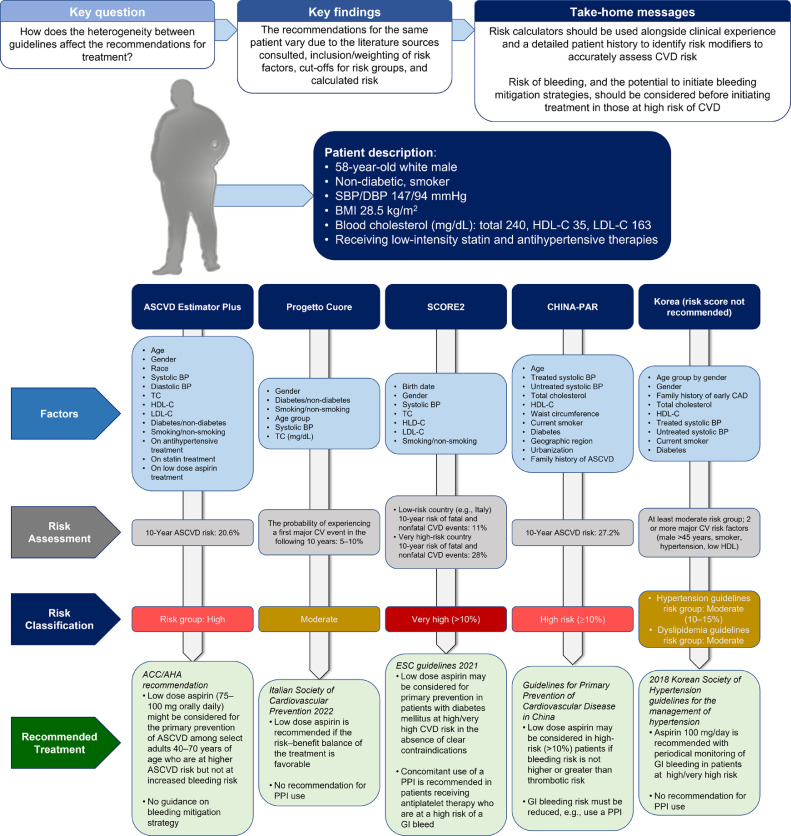
**Central illustration** Heterogeneity of recommendations for initiation of low dose aspirin use for primary cardiovascular prevention across five major clinical management guidelines. This scenario depicts the recommendations from primary prevention guidelines (from top to bottom) for Italy, South Korea, the USA, China, and Europe for the same hypothetical patient with several CVD risk factors. These contribute to a current 10-year ASCVD estimated risk of 24.7% according to the ASCVD Risk Estimator Plus. The recommendations are based on the patient's high risk of a CV event and the guidance, where given, regarding the benefit:risk balance of low dose aspirin. ASCVD, atherosclerotic cardiovascular disease; BMI, body mass index; CAC, coronary artery calcium; CVD, cardiovascular disease; DBP, diastolic blood pressure; GI, gastrointestinal; HDL-C, high-density lipoprotein cholesterol; LDL-C, low-density lipoprotein cholesterol; PPI, proton pump inhibitor; SBP, systolic blood pressure; T2DM, type 2 diabetes mellitus; TC, total cholesterol.

We conducted a review of the most recent guidelines for primary prevention used in the authors’ regions/countries—Europe, Italy, the USA, China, and South Korea—with the aim of establishing the commonalities and differences between risk factor targets, risk modifiers, and treatment approaches, with a specific focus on the degree to which these guidelines help to identify patients who are most likely to benefit from primary prevention with low dose aspirin therapy (Fig. 1).

## 2 The history and diversity of risk assessment in the primary prevention of cardiovascular disease

The Framingham 10-year Coronary Heart Disease (CHD) risk prediction equation (Framingham-CHD) was the first risk assessment tool to be developed and was based on a prospective single-center study of the Framingham Heart Study Community Cohort in the USA [12]. This was updated with guidance on dyslipidemia in 2001 to the National Cholesterol Education Program Adult Treatment Panel III (NCEP ATP-III) assessment tool [13] and then to the Framingham General CVD Risk Profile tool, which included assessments for the 10-year risk of CHD, cerebrovascular events, peripheral artery disease, and heart failure [14]. However, limitations in these tools were recognized, such as their basis on historical data that were limited to White patients and the initial exclusion of ischemic stroke. Introduced in 2013 [15], the pooled cohort equations (PCE), which include specific risk assessments for African Americans, are integral to the ASCVD Risk Estimator Plus calculator currently recommended for the estimation of 10-year ASCVD risk for asymptomatic adults aged 40–75 years in the USA [16] (Table 1).

**Table 1:** Comparison between risk calculators in the USA, Europe, Italy, China, and South Korea.

	**CV risk calculator**				
	**ASCVD Risk Estimator Plus**	**SCORE2**	**Progetto Cuore**	**China-PAR**	**Korean Risk Prediction Model**
**Outputs: 10-year risk of:**					
Fatal and nonfatal CVD	X	✓	X	X	X
Fatal CHD	✓	✓	✓	✓	✓
Nonfatal CHD	X	X	✓	X	✓
Fatal MI	X	X	X	✓	✓
Nonfatal MI	✓	✓	X	✓	✓
Fatal stroke	✓	✓	✓	✓	✓
Nonfatal stroke	✓	✓	✓	✓	✓
Revascularization	X	X	✓	X	X
Sudden death	X	X	✓	X	✓
**Risk factors included in calculator**					
Region	X	✓	X	✓	X
Ethnicity	✓	X	X	X	X
Specific location	X	✓	X	X	X
Sex	✓	✓	✓	✓	✓
Age	✓	✓	✓	✓	✓
Smoker	✓	✓	✓	✓	✓
Previous smoker	X	X	X	X	✓
Diabetes	✓	✓	✓	✓	✓
Duration of diabetes	X	X	X	X	X
HbA_1c_	X	X	X	X	X
Family history	X	X	X	✓	X
BMI (or height/ weight/waist)	X	X	X	✓ (waist)	X
Hypertension	✓	X		✓	✓
Systolic BP	✓	✓	✓	✓	✓
Antihypertensive treatment	✓	✓	✓	✓	✓
Total cholesterol	✓	✓	✓	✓	✓
HDL-C	✓	✓	✓	✓	✓
LDL-C	✓	X	X	X	✓
Triglycerides	X	X	X	X	X
Lipid-lowering treatment	✓	X	X	X	X
CAC	X	X	X	X	X

ASCVD, atherosclerotic cardiovascular disease; BMI, body mass index; BP, blood pressure; CAC, coronary artery calcium; CHD, coronary heart disease; China-PAR, Prediction for ASCVD Risk in China; CVD, cardiovascular disease; HbA_1c_, glycated hemoglobin; HDL-C, high-density lipoprotein-cholesterol; LDL-C, low-density lipoprotein-cholesterol; MI, myocardial infarction; SCORE, Systematic COronary Risk Evaluation.

Based on the Framingham risk assessment tools, the Systematic COronary Risk Evaluation (SCORE), a comprehensive risk assessment model, was developed for both high-risk and low-risk regions of Europe [17]. Updated, recalibrated country-specific versions of SCORE risk charts have been published for all European countries [18]. The recently published SCORE2 has several advantages over the original SCORE model: it is based on the most contemporary and representative datasets on CVD in all European countries, grouped into 4 risk regions (low, moderate, high, and very high risk) according to their most recently reported World Health Organization (WHO) age- and sex-standardized overall CVD mortality rates, and provides estimates for both fatal and nonfatal CV events [19,20] (the previous focus on fatal events alone may have underestimated total CVD burden) [21]. The SCORE2-Older Persons (SCORE2-OP) risk model estimates 5- and 10-year risk of CVD in individuals aged >70 years in the 4 risk regions [19,20]. The Progetto Cuore score tailored the SCORE model for regions in Italy, adding 2 additional risk factors (diabetes and hypertension) and a new endpoint (nonfatal CV events) [22]. In China, the risk assessment model for risk factors and ischemic CVD events was established in 2003 [23]. Subsequent models include the Prediction for ASCVD Risk in China (China-PAR) equations [24]. Among these models, the ASCVD Risk Assessment Process Flow Chart for Chinese Adult Population is currently the most widely used for primary prevention [25]. In South Korea, the Korean Risk Prediction Model (KRPM) was developed using data from the Korean Heart Study cohort of over 200,000 adults aged 40–79 years who were free from ASCVD at baseline [26]. The KRPM improved ASCVD risk prediction in this population compared with the PCE, which overestimated this risk by approximately twofold [27]. The lack of easy-to-use tools that incorporate the KRPM, however, has inhibited its adoption into local guidelines for risk assessment. In addition, there have been concerns and some evidence that further recalibration of this model is required to better predict outcomes in clinical practice in South Korea [28].

## 3 Identification of high-risk patients in the primary prevention population in China, the USA, South Korea, Italy, and Europe

The presence of modifiable risk factors such as tobacco use, poor diet, excess body weight or obesity, hypertension, dyslipidemia, and diabetes mellitus (DM) are key indicators of CV risk. However, because the development of ASCVD is usually the result of multiple, interacting factors, assessment of total CVD risk is recommended [29]. Across the 5 guidelines for primary prevention reviewed here (Table 2) [8,16,25,[30], [31], [32]] the categories of risk severity differ, as do the definitions used for each category. Only the European Society of Cardiology (ESC)/European Society of Hypertension (ESH) and Chinese guidelines include a very high risk category. Patients could be classified as high risk with *a*≥ 20% 10-year CVD risk (American College of Cardiology [ACC]/American Heart Association [AHA] 2019 and the Progetto Cuore), *a*≥ 10% 10-year CVD risk (China-PAR), or *a*≥ 5% to <10% 10-year risk (SCORE), although the outputs from the respective risk calculators also differ (Table 1).

**Table 2:** Risk categories for cardiovascular disease according to primary prevention guidelines in the USA, Europe, Italy, China, and South Korea.

**CVD risk**	**US 2019**[16]	**ESC 2021**[8]	**Italy 2018**[33]	**China 2020**[25]	**South Korea 2018** [31,32]*
Very high	Not categorized	Apparently healthy subjects with 10-year ASCVD risk (according to age) of: • ≥15% (≥70 years) • ≥10% (50–69 years) • ≥7.5% (<50 years)	Not applicable	Documented ASCVD	2018 Dyslipidemia guidelines • CAD • PAD • atherosclerotic ischemic stroke • TIA 2018 Hypertension guidelines • Not categorized
High	≥20% 10-year ASCVD risk^†^	Apparently healthy subjects with 10-year ASCVD risk (according to age) of: • 7.5% to <15% (≥70 years) • 5% to <10% (50–69 years) • 2.5% to <7.5% (<50 years)	≥20% 10-year CV risk (first major CV event)	Subjects with ≥10% 10-year ASCVD risk: • Diabetes and ≥40 years • LDL-C ≥ 4.9 mmol/L (189.5 mg/dL) or TC ≥7.2 mmol/L (278.4 mg/dL) • CKD stages 3–4 • High-normal BP + 3 risk factors • Grade 1 high BP + 2 risk factors • Grade 2–3 high BP + 1 risk factor • Moderate CVD risk + ≥2 of the 5 following risk factors: - Grade 2–3 high BP - Non-HDL-C ≥ 5.2 mmol/L (201.1 mg/dL) - HDL-C <1.0 mmol/L (38.7 mg/dL) - BMI ≥28 kg/m^2^ - Smoker	2018 Dyslipidemia guidelines • carotid artery disease • abdominal aortic aneurysm • DM 2018 Hypertension guidelines: ≥15% 10-year CVD • DM complicated by subclinical organ damage or CVD • CVD • CKD OR • Grade 1 hypertension with ≥3 major risk factors OR • Grade 2 hypertension with 1–2 risk factors
Intermediate	• ≥7.5% to <20% 10-year ASCVD risk • Use CAC score to guide decisions	Not categorized	Not applicable	Not categorized	Not categorized
Moderate	Not categorized	• SCORE is ≥1% and <5% at 10 years. Many middle-aged subjects belong to this category.	≥3 to <20% 10-year CV risk	Subjects with 5–9% 10-year ASCVD risk: • normal BP + hypercholesteremia + 2–3 risk factors • Grade 1 high BP + hypercholesteremia + 1 risk factor • Grade 2 high BP + normal cholesterol + 2 risk factors	2018 Dyslipidemia guidelines • ≥2 major risk factors 2018 Hypertension guidelines: ≥10% to <15% 10-year CVD • Grade 2 hypertension • Grade 1 hypertension with 1–2 risk factors • prehypertension with ≥3 major risk factors
Low-to-moderate	Not categorized	• <7.5% (≥70 years) • <5% (50–69 years) • <2.5% (<50 years)	Not categorized	Not categorized	Not categorized
Borderline risk	• 5% to <7.5% 10-year ASCVD risk • Use risk-enhancing factors, such as CAC score to guide decisions	Not categorized	Not applicable	Not categorized	Not categorized
Low risk	<5% 10-year ASCVD risk^†^	SCORE <1%	<3% 10-year CV risk	Subjects with <5% 10-year ASCVD risk: • normal BP + normal cholesterol level + ≤3 risk factors • high BP + normal cholesterol + ≤1 risk factor • high BP + hypercholesterolemia + no other risk factor	2018 Dyslipidemia guidelines • ≤1 major risk factor 2018 Hypertension guidelines: 5–<10% 10-year CVD • Grade 1 hypertension • prehypertension with 1–2 risk factors

*References: Dyslipidemia guidelines [31]; hypertension guidelines [32]. ^†^ASCVD Risk Estimator Plus outcome.

ACS, acute coronary syndrome; AMI, acute myocardial infarction; ASCVD, atherosclerotic cardiovascular disease; BMI, body mass index; BP, blood pressure; CAC, coronary artery calcium; CAD, coronary artery disease; CKD, chronic kidney disease; CV, cardiovascular; CVD, cardiovascular disease; DM, diabetes mellitus; ESC, European Society of Cardiology; ESH, European Society of Hypertension; GFR, glomerular filtration rate; HDL-C, high-density lipoprotein-cholesterol; LDL-C, low-density lipoprotein-cholesterol; PAD, peripheral artery disease; SCORE, Systematic Coronary Risk Evaluation; TC, total cholesterol; TIA, transient ischemic attack.

According to the ASCVD Risk Assessment Process Flow Chart for Chinese Adult Population [25], patients with diabetes aged ≥40 years, with low-density lipoprotein-cholesterol (LDL-C) ≥4.9 mmol/L (189 mg/dL) or total cholesterol (TC) ≥7.2 mmol/L (278.4 mg/dL), or with chronic kidney disease (CKD) stage 3–4, have a high risk of ASCVD (defined as predicted 10-year CVD risk ≥10%). In other individuals, the 10-year ASCVD risk should be estimated by considering age, blood pressure, LDL-C or TC levels, high-density lipoprotein-cholesterol (HDL-C) level, smoking (yes/no), body mass index (BMI), and family history of CVD.

Since 2003, European guidelines on CVD prevention in clinical practice have recommended the SCORE system. At the population level, however, absolute event rates vary across different subgroups. Thus, the SCORE system has been adapted to suit populations in low-, high-, and very high-risk European countries, but not to address the different ethnic groups within those countries [29]. Similarly, performance of the PCE in diverse population subgroups from outside the USA is highly variable, reflecting the heterogeneous nature of the populations in terms of the prevalence of risk factors and inherent hazards for ASCVD. Thus, the PCE systematically underestimate risk in patients from some racial/ethnic groups, those with lower socioeconomic status, or with chronic inflammatory diseases, and over-predict risk in patients with higher socioeconomic status or those receiving preventive health care and follow-up [9].

The under- or overestimation of risk illustrates two important aspects of CV risk prediction. Firstly, risk prediction equations developed in one population cannot be satisfactorily applied to other populations, or even used in the same country years after they were originally developed, because of changes in average risk factor levels and disease risks [34]. Secondly, factors other than the traditional CVD risk factors may significantly alter the risk of CVD development in subsets of patients.

The applicability of a risk prediction score can be improved by recalibration of the prediction model according to the average risk factor levels and disease risks of the target population. The Globorisk CVD risk score, for example, can be recalibrated and updated for use in different countries and years with routinely available information. This risk prediction equation can be used to predict 10-year risk of fatal and nonfatal CVD in 182 countries worldwide, with and without laboratory-based measurements [34,35], and allows for the variation in CV risk with sex and age [36].

Apart from the conventional major CV risk factors included in risk prediction charts, several other factors modify or enhance risk and can be relevant for assessing total CVD risk (Table 3). Assessment of additional risk factors is recommended to improve risk classification and, if feasible, is of value where an individual's risk borders a risk category threshold [16,29,37].

**Table 3:** Risk-enhancing factors and risk modifiers that should be considered in the assessment of cardiovascular risk in the primary prevention population primary prevention guidelines in the USA, Europe, Italy, China, and South Korea.

**Risk-enhancing or -modifying factor**	**ACC/AHA 2019**	**ESC 2021**	**Italy**	**China**	**South Korea**
Socioeconomic status, social isolation, or lack or social support, domestic abuse/violence		✓	✓		
Family history of premature ASCVD	✓	✓	✓	✓	✓
Primary hypercholesterolemia	✓ (LDL-C, 160–189 mg/dL [4.1–4.8 mmol/L]; non–HDL-C 190–219 mg/dL [4.9–5.6 mmol/L])			✓	
Metabolic syndrome	✓				✓
Changes in renal function/CKD	✓	✓	✓		✓
Chronic inflammatory conditions	✓	✓	✓		
History of premature menopause (before age 40 years)	✓	✓			
History of pregnancy-associated conditions that increase later ASCVD (e.g., preeclampsia)	✓				
High-risk race/ethnicity	✓	✓			
Post-traumatic stress			✓		
Lipids/biomarkers associated with increased ASCVD risk	✓			✓	
Persistent primary hypertriglyceridemia (≥175 mg/dL, nonfasting)	✓			✓	✓
Elevated hsCRP (≥2.0 mg/L)	✓		✓	✓	
Elevated Lp(a) (≥50 mg/dL or ≥125 nmol/L)	✓			✓	✓
Elevated apoB (≥130 mg/dL)	✓			✓	✓
ABI <0.9	✓	✓	✓	✓	
BNP/NT-pro-BNP			✓		
BMI and central obesity		✓	✓	✓	
CAC score	✓	✓	✓	✓	✓
Atherosclerotic plaque documented by carotid artery scanning		✓	✓	✓	✓
Increased intima-media thickness of carotid arteries			✓	✓	✓
Nonobstructive coronary artery stenosis (<50%)				✓	
Left ventricular hypertrophy			✓	✓	

ABI, ankle–brachial index; ACC, American College of Cardiology; AHA, American Heart Association; apoB, apolipoprotein B; ASCVD, atherosclerotic cardiovascular disease; BMI, body mass index; BNP, B-type natriuretic peptide; CAC, coronary artery calcium; CKD, chronic kidney disease; ESC, European Society of Cardiology; ESH, European Society of Hypertension; hsCRP, high-sensitivity C-reactive protein; LP(a), lipoprotein a; NT-pro-BNP, N-terminal pro-B-type natriuretic peptide.

Use of the coronary artery calcium (CAC) score or other arterial imaging, including carotid ultrasound, has been proposed to help guide decisions about preventive interventions in selected patients. Several features of the CAC score underpin its potential value as a tool for ASCVD risk stratification. CAC detection by computed tomography (CT) is highly sensitive, pathognomonic of atherosclerotic plaque, and CAC burden correlates strongly with total coronary plaque. CAC scoring can be performed with any modern CT scanner, and is rapid, exposes the patient to only a low radiation dose, and allows simple semi-automated interpretation that, using standardized CT parameters, is consistent worldwide [38]. Importantly, CAC scoring may identify a distinct group of patients: those with advanced subclinical atherosclerosis whose place in the chronic coronary syndrome continuum may overlap the traditional boundaries of primary and secondary prevention [39]. For patients with type 2 diabetes mellitus (T2DM), the severity of CAC appears to be a stronger clinical prognostic indicator than conventional measures of disease severity, such as insulin use or glycemic control. The addition of CAC score to global risk assessment is associated with significantly improved risk classification in patients with T2DM and no known CVD; the absence of CAC is associated with low ASCVD and CHD risk, even in those with diabetes duration longer than a decade [39]. Thus, CAC testing appears to have considerable utility as a decision aid for CV risk reduction interventions. Notably, in patients younger than 70 and without overt CVD or high bleeding risk, modeling studies suggest that detection of CAC ≥100, and particularly CAC ≥400, could identify those likely to gain net benefit from low dose aspirin therapy [40].

CAC score is identified as a risk modifier in most guidelines and its use to help guide decisions about statin or low dose aspirin treatment in patients with intermediate 10-year ASCVD risk (≥7.5% to <20%) is recommended in two of the primary prevention guidelines reviewed here—the 2019 ACC/ AHA guideline and the 2021 ESC guidelines [8,16]—as well as the US Combined Societies’ 2018 guideline on the management of blood cholesterol [37] and the new US National Lipid Association (NLA) recommendations for primary prevention therapy according to CAC score, which include a moderate strength recommendation for the reasonable use of low dose aspirin in patients with CAC ≥100 and no bleeding-related contraindications for such therapy [41].

Ultrasound assessment of carotid plaque burden can also enhance ASCVD prediction using traditional risk factors in asymptomatic adults [42,43]. Carotid artery color Doppler ultrasound is a low-cost, non-invasive, simple procedure that is radiation-free and repeatable [44]. Globally, the burden of carotid atherosclerosis is substantial [45], and systematic analysis of stroke rates reported in observational cohort studies shows that the risk of stroke is highly correlated with the degree of asymptomatic carotid stenosis [46]. Of the guidelines reviewed here, only the European guidelines include recommendations for carotid artery plaque assessment using ultrasonography as a risk modifier in CV risk prediction. This may be considered in some cases when a CAC score is not feasible [8], and should be considered for asymptomatic patients with DM [47]. Interestingly, the use of ultrasound-based imaging of subclinical carotid atherosclerosis to inform primary care physicians and patients improves CV risk reduction through better adherence to primary prevention measures [48].

### 3.1 Other risk modifiers

Numerous other factors have been identified as modifying or enhancing CV risk, but are not included in risk estimation models. The significance of CKD as a CV risk factor is well recognized in primary prevention guidelines [8,16,29]. The European guidelines recognize the importance of decreasing estimated glomerular filtration rate (eGFR) and, independently, increasing albuminuria as indicators of worsening renal impairment and increasing CVD risk, but note that the use of CAC score may be useful to stratify CV risk in patients with CKD [8]. The Italian Progetto Cuore similarly notes that changes in renal function may be integrated into the risk evaluation [30]. For patients with diabetes, the 2019 ESC guidelines recommend stratification of CKD according to both eGFR and albuminuria [47]. Individuals with CKD stages 3–4 are directly categorized as high-risk ASCVD patients by the Chinese guidelines [25].

An increasing number and variety of other clinical, behavioral, and environmental factors are being recognized as affecting CV risk. Nonalcoholic fatty liver disease, for example, is associated with increased risk of CV events, regardless of traditional risk factors such as diabetes and dyslipidemia [49].

### 3.2 Are we underestimating the cardiovascular risk in patients with diabetes?

Individuals with diabetes have accelerated atherosclerosis in different vascular territories [50], with higher atheroma volume, more atherosclerotic plaque, and much narrower coronary lumens than those without diabetes [51]. Thromboxane A_2_-dependent platelet activation has been shown to be at least as high in patients with preclinical and early diabetes as in those with T2DM [52]. The risk of negative CV outcomes in patients with T2DM after a decade, those with target organ damage, or those with 3 or more CV risk factors seems to be equivalent to patients with coronary artery disease (CAD). Based on this concept, patients with diabetes are classified into 3 categories: very high, high, and moderate CV risk. In a cross-sectional study of a primary care database in Catalonia, Spain (SIDIAP) that included 373,185 adults with a diagnosis of T2DM, a significant proportion (36.4%, 95% confidence interval [CI]: 36.1–36.7) exhibited very high CV risk that classified them as “CAD-equivalent” patients [53]. Risk of CVD is increased twofold in patients with diabetes in general [53] and is already increased in prediabetes [54].

The current American Diabetes Association (ADA) Standards of Care in Diabetes recommend use of the ACC/AHA ASCVD Risk Estimator Plus for CV risk calculation in patients with T2DM but acknowledge that this does not account for the duration of diabetes or the presence of diabetes complications, such as albuminuria [55]. The 2019 ESC in collaboration with the European Association for the Study of Diabetes (EASD) guidelines do not recommend the use of risk scores developed for the general population in those with diabetes but provide specifically designed risk stratification, which excludes low-risk patients and, rather than the inclusion of traditional risk factors, focuses on duration of diabetes and target organ damage [47]. Better identification of early target organ damage would improve risk stratification of patients with prediabetes and T2DM, and allow measures to target T2DM-related factors and precursors of CV risk more aggressively.

The 2019 ACC/AHA guideline [16] provides a class IIa recommendation for initiating metformin as first-line pharmacotherapy at diagnosis of T2DM to reduce ASCVD risk in addition to improving glycemic control. For patients with T2DM and additional CV risk factors who require glucose-lowering therapy despite metformin (and lifestyle modifications), initiation of a sodium-glucose cotransporter 2 (SGLT-2) inhibitor or a glucagon-like peptide 1 receptor agonist (GLP-1RA) may be reasonable to improve glycemic control and reduce CVD risk. The use of low dose aspirin, which would not be expected to have reduced efficacy with concomitant use of these glucose-lowering agents, at the time of diagnosis of T2DM seems reasonable if there is no excess bleeding risk [56] but is not specified in these guidelines. In contrast, the ADA Standards of Care in Diabetes recommends the use of low dose aspirin for primary prevention in men and women aged ≥50 years with diabetes and at increased CV risk (at least one additional major risk factor) [55]. The 2021 ESC and 2019 ESC/EASD guidelines recommend that low dose aspirin be considered for primary prevention in patients with DM and at least high CV risk, which includes all except those with well-controlled DM of <10 years duration without target organ damage or any other major CV risk factor. Noting that asymptomatic patients with DM can have a significant atherosclerotic burden, the ESC/EASD guidelines include a class IIb recommendation for CAC scoring by CT in such patients with at least moderate risk [47]. A joint position paper of the Italian Cardiology (SIC) and Italian Diabetes (SID) Societies on CV risk management in T2DM does not make any specific recommendation for or against the use of low dose aspirin in primary prevention [57]. The most recent Chinese T2DM guideline recommends that aspirin can be used for primary prevention in T2DM patients aged ≥50 years without high bleeding risk and with at least one of the following risk factors: family history of premature ASCVD, hypertension, dyslipidemia, smoking, or CKD/proteinuria [58].

### 3.3 Risk remains despite statin therapy or due to poor adherence to statin therapy

The evidence supporting the use of low dose aspirin in primary prevention has been criticized as being mostly based on older clinical trials that included patients with higher smoking rates and lower use of antihypertensive agents and statins [59]. However, more than 80% of high-risk patients do not achieve recommended LDL-C targets, partly due to the use of insufficient starting doses of statins and the low adherence/high discontinuation rate of chronic statin treatment. Statin adherence rates of just 25% in primary prevention have been reported in the USA [60]. The Chinese consensus statement notes that patients who are unable to take other primary prevention measures (such as statins) may need low dose aspirin more often [61]. A cross-sectional, multicenter observational study to assess control of CV risk factors among Chinese adults with T2DM found that only 5054 of 25,454 patients (19.9%) were on statin treatment, and none of the 14,766 patients without dyslipidemia were taking any lipid-modulating drugs [62]. These treatment proportions are significantly lower than the 75.3% of patients with DM using statin therapy in the ASCEND trial [63].

## 4 Recommendations for the use of low dose aspirin in the primary prevention of cardiovascular disease in China, the USA, South Korea, Italy, and Europe

Low dose aspirin is recommended or may be considered for use in primary prevention in selected populations in all the countries/regions included in this review. This includes Europe, for which the ESC guidance—previously a notable exception [29]—now notes that aspirin may be considered for patients without established ASCVD at high or very high CVD risk, in addition to formally recommending its use for the majority of patients with DM [8] (Tables 4 and 5). The US Preventive Services Task Force (USPSTF) has recently updated its recommendation for low dose aspirin use, now stating that the decision to initiate low dose aspirin for the primary prevention of CVD in adults aged 40–59 years who have a ≥ 10% 10-year CVD risk should be an individual one and recommending against initiating low dose aspirin use for the primary prevention of CVD in adults aged ≥60 years [64].

**Table 4:** Summary of recommendations for and against the use of low dose aspirin in the USA, Europe, Italy, China, and South Korea.

**Guideline (reference)**	**Recommendation(s) for low dose aspirin**	**Class/strength of recommendation**	**Level/ quality of evidence**
2019 ACC/AHA guideline on the primary prevention of CVD [16]	Low dose aspirin (75–100 mg/d orally) might be considered among select adults 40 to 70 years of age who are at higher ASCVD risk but not at increased bleeding risk	IIb	A
	Low dose aspirin (75–100 mg/d orally) should not be administered on a routine basis for the primary prevention of ASCVD among adults >70 years of age	III	B-R
	Low dose aspirin (75–100 mg/d orally) should not be administered for the primary prevention of ASCVD among adults of any age who are at increased risk of bleeding	III	C-LD
2022 US Preventive Services Task Force Recommendation Statement: Aspirin Use to Prevent Cardiovascular Disease [64]	The decision to initiate low dose aspirin use for the primary prevention of CVD in adults aged 50–59 years with a ≥ 10% 10-year CVD risk should be an individual one	C	Moderate
	Do not initiate aspirin for the primary prevention of CVD in adults ≥60 years	D	Moderate
2021 ESC guidelines on cardiovascular disease prevention in clinical practice [8]	In selected patients without established ASCVD at high or very high CVD risk, the benefits of aspirin outweigh the risks	Not provided	Not provided
2016 European guidelines on CVD prevention in clinical practice [29]	Antiplatelet therapy is not recommended in individuals without CVD due to the increased risk of bleeding	III	B
	Antiplatelet therapy is not recommended for people with DM who do not have CVD	III	A
2019 Chinese expert consensus statement on aspirin application in primary prevention of CVD [61]	The following ASCVD high-risk groups may consider taking low dose aspirin (75–100 mg/day) for primary prevention: adults aged 40–69 years, if the 10-year expected risk of ASCVD is ≥10% for their initial risk assessment, and there are still ≥3 major risk factors that remain poorly controlled or difficult to change after active treatment intervention. The main risk factors include: • Hypertension, diabetes, dyslipidemia (TC ≥6.2 mmol/L [239.7 mg/dL], or LDL ≥4.1 mmol/L [158.5 mg/dL], or HDL <1.0 mmol/L [38.7 mg/dL]), smoking, family history of early onset CVD, obesity, CAC score ≥100, or coronary artery stenosis <50%	IIb	A
	The following populations are not recommended to take aspirin for primary prevention of ASCVD:		
	Population aged ≥70 years or <40 years	III	B
	Population at high risk of bleeding	III	C
	Patients whose risk of bleeding was assessed to be greater than the risk of thrombosis	III	C
2018 Joint consensus document on CVD prevention in Italy [33]	Recommended for subjects with a risk of major CV events ≥2/100 patient-years (equivalent to a SCORE risk of 7–10% at 10 years), especially if male and aged 50–60 years. Risk:benefit ratio for aspirin use in primary prevention • Yes to aspirin: ∘ Cancer risk: age, sex, smoking, family history, precancerous lesion, genetic syndromes and polymorphisms, dietary and lifestyle habits, exposure to radiation ∘ CV risk: age, male sex, hypertension, dyslipidemia, obesity, smoking, dietary and lifestyle habits, family history, menopause • No to aspirin: ∘ Bleeding risk: age, previous bleedings, liver or kidney failure, peptic ulcer and GI disorders, concomitant use of NSAID or anticoagulant therapy, previous stroke, severe comorbidities	Not provided	Not provided
2017 Chinese CVD prevention guide [65]	Low dose aspirin is recommended for • 10-year ASCVD risk ≥10% • Diabetic patients, age ≥50 years, with at least one major risk factor • Hypertensive patients with BP <150/90 mmHg, accompanied by ≥3 major risk factors • Patients with CKD (eGFR 30–45 mL/min/1.73 m^2^) • Patients other than above with ≥4 risk factors	Not provided	Not provided
2020 Guideline for primary prevention of CVD in China [25]	Low dose aspirin might be considered in adults aged 40–70 years who are at high ASCVD risk but not at increased bleeding risk, with at least one risk-enhancing factor (e.g., CAC >100, carotid plaque)	IIb	A
2018 Korean Society of Hypertension guidelines for the management of hypertension: Part II – diagnosis and treatment of hypertension [32]	The role of aspirin for primary prevention remains a matter of debate, leaning towards the negative side Low dose aspirin (100 mg) can be prescribed to patients in high-risk groups in order to reduce the risk of CVD, e.g., hypertensive patients with CKD [60] Antiplatelet agents should be administered after BP is controlled, and patients should be checked periodically for GI bleeding	Not provided	Not provided

ASCVD, atherosclerotic cardiovascular disease; BMI, body mass index; BP, blood pressure; CAC, coronary artery calcium; CKD, chronic kidney disease; CV, cardiovascular; CVD, cardiovascular disease; DM, diabetes mellitus; eGFR, estimated glomerular filtration rate; GI, gastrointestinal; HDL-C, high-density lipoprotein-cholesterol; LDL-C, low-density lipoprotein-cholesterol; MI, myocardial infarction; NSAID, nonsteroidal anti-inflammatory drug; SCORE, Systematic COronary Risk Evaluation; TC, total cholesterol.

**Table 5:** Summary of recommendations for and against the use of low dose aspirin in patients with diabetes mellitus in the USA, Europe, Italy, China, and South Korea.

**Guideline (reference)**	**Low dose aspirin recommendations**	**Class/strength of recommendation**	**Level/ quality of evidence**	**Evidence base for key recommendations (reference)**
2019 ESC guidelines on diabetes, prediabetes, and CVDs [47]	Aspirin (75–100 mg/day) for primary prevention may be considered in patients with DM at very high/high risk in the absence of clear contraindications (GI bleeding, peptic ulceration within the previous 6 months, active hepatic disease, or history of aspirin allergy)	IIb	A	3
	Aspirin for primary prevention is not recommended in patients with DM at moderate CV risk	III	B	
	Concomitant use of a PPI is recommended in patients receiving aspirin monotherapy, DAPT, or oral anticoagulant monotherapy who are at high risk of GI bleeding	IIa	A	105, 106
2020 American Diabetes Association. 10. Cardiovascular Disease and Risk Management: Standards of Medical Care in Diabetes – 2020 [55]	Aspirin therapy (75–162 mg/day) may be considered as a primary prevention strategy in those with diabetes who are at increased CV risk, after a comprehensive discussion with the patient on the benefits versus the comparable increased risk of bleeding		A	
2016 European guidelines on CVD prevention in clinical practice [29]	Antiplatelet therapy is not recommended for people with DM who do not have CVD	III	A	107
2021 ESC guidelines on cardiovascular disease prevention in clinical practice [8]	In patients with DM at high or very high CVD risk, low dose aspirin may be considered for primary prevention in the absence of clear contraindications	IIb	A	3, 43, 108
2020 Società Italiane di Cardiologica e Società Italiane di Diabetologia joint document [57]	• Antiplatelet therapy is not recommended for people with DM who do not have CVD • In diabetic patients with multiple risk factors for ASCVD, aspirin use in primary prevention must be evaluated on an individual basis after accurate clinical judgment	I I	B C	1, 43, 105, 109–115
Guideline for prevention and treatment of type 2 diabetes in China (2020 edition) [58]	Aspirin therapy as a primary prevention strategy can be recommended in diabetic patients with high CVD risk; that is, those aged ≥50 years with at least one additional major risk factor (family history of premature ASCVD, hypertension, dyslipidemia, smoking, or CKD/albuminuria) without high bleeding risk	IIb	Not provided	3, 4, 5, 11, 43, 52, 58, 86, 116
2019 Clinical practice guidelines for type 2 diabetes mellitus in Korea [66]	Aspirin (100 mg daily) may be considered for primary prevention in patients with DM at high CV risk, if they do not have high bleeding risk	IIb	C	52

ASCVD, atherosclerotic cardiovascular disease; CKD, chronic kidney disease; CV, cardiovascular; CVD, cardiovascular disease; DAPT, dual antiplatelet therapy; DM, diabetes mellitus; GI, gastrointestinal; PPI, proton pump inhibitor.

It is important to note that most recommendations for the use of low dose aspirin in selected patients for primary prevention of CVD have a IIb level of evidence, reflecting the lack of high-quality randomized controlled trials in this area, with residual uncertainty. The specific guidance for aspirin use, and, if given, for concomitant proton pump inhibitor (PPI) use, varies and likely reflects the variation in the evidence selected for evaluation (Table 6). While the 2021 ESC guidelines [8] note that CAC scoring can help reclassify CV risk, for example, the ACC/AHA [16] guidelines provide specific CAC score values for CV risk reclassification and initiation of statin therapy.

**Table 6:** Publications cited as evidence for recommendations/guidance on the use of low dose aspirin for primary prevention of cardiovascular disease in the USA, Europe, Italy, China, and South Korea.

**Publication [reference number]**	**Acronym**	**Patients with DM**	**2019 ACC/AHA**	**USPSTF 2022**	**2021 ESC**	**Italian**	**Chinese**	**South Korean**
**Guidelines**								
Arnett 2019 ACC/AHA Guidelines [16]	ACC/AHA			✓				
Piepoli MF, et al. Eur Heart J 2016;37:2315–81 [29]	ESC		✓			✓		
Pearson TA, et al. Circulation 2002;106:388–91 [67]	AHA		✓			✓	✓	
Pignone M, et al. Circulation 2010;121:2694–701 [68]	ADA		✓			✓		
Halvorsen S, et al. J Am Coll Cardiol 2014;64:319–27 [69]	ESC		✓		✓	✓		
Bibbins-Domingo K; US Preventive Services Task Force. Ann Intern Med 2016;164:83645 [70]	USPSTF		✓	✓		✓	✓	
Rhee EJ, et al. Korean J Intern Med 2019;34:723–71 [31]								✓
Lee HY, et al. Clinical Hypertens 2019;25:20 [32]								✓
Kim MK, et al. Diabetes Metab J 2019;43:398–406 [66]								✓
Cosentino F, et al. Eur Heart J 2020;41:255–323 [47]	ESC				✓			
Visserin FLJ, et al. Eur Heart J 2021; 42:3227–337 [8]	ESC				✓			
**Meta-analyses and reviews**								
Antithrombotic Trialists’ Collaboration. Lancet 2009;373:1849–60 [71]	ATC		✓		✓	✓	✓	
Guirguis-Blake JM, et al. Ann Intern Med 2016;164:804–13 [72]	USPSTF		✓					
Garcia Rodriguez LA, et al. PLoS ONE 2016;11:e0160046 [73]			✓					
Capodanno D, Angiolillo DJ. Circulation 2016;134:1579–94 [74]			✓					
Raju N, et al. Am J Med 2016;129:e35–6 [75]			✓					
Whitlock EP, et al. Ann Intern Med 2016;164:826–35 [76]	USPSTF		✓					
Mora S, Manson JE. JAMA Intern Med 2016;176:1195–204 [77]			✓				✓	
Ridker PM. N Engl J Med 2018;379:1572–4 [78]			✓					
Rothwell PM, et al. Lancet 2018;392:387–99 [79]			✓	✓				
De Berardis G, et al. BMJ 2009;339:b4531 [80]		✓		✓	✓	✓		
Lotrionte M, et al. Prog Cardiovasc Dis 2016;58:495–504 [81]			✓					
Joint Task Force for Guideline on the Assessment and Management of Cardiovascular Risk in China 2019. Zhonghua Yu Fang Yi Xue Za Zhi 2019;53:13–35 [82]							✓	
Geriatrics Branch of Chinese Medical Association 2017; Editorial Board of Chinese Journal of Internal Medicine; Editorial Board of Chinese Journal of Geriatrics. Zhonghua Nei Ke Za Zhi 2017;56:68–80 [83]							✓	
Task Force on Chinese Guidelines for the Prevention of Cardiovascular Diseases (2017); Editorial Board of Chinese Journal of Cardiology. Zhonghua Xin Xue Guan Bing Za Zhi 2018;46:10–25 [65]			✓				✓	
Abdelaziz HK, et al. J Am Coll Cardiol 2019;73:2915–29 [84]					✓			
Zheng SL, Roddick, AJ. JAMA 2019;321:277–87 [85]					✓			
Mahmoud AN, et al. Eur Heart J 2019;40:607–17 [86]					✓			
Berger JS, et al. JAMA 2006;295:306–31 [87]					✓			
Yusuf S, et al. N Engl J Med 2021;384:216–28 [88]					✓			
Seidu S, et al. Cardiovasc Diabetol 2019;18:70 [89]					✓			
Contemporary RCTs*								
ASCEND Study Collaborative Group. N Engl J Med 2018;379:1529–39 [4]	ASCEND	✓	✓		✓		✓	
Belch J, et al. BMJ 2008;337:a1840 [90]	POPADAD	✓	✓			✓		
Fowkes FGR, et al. JAMA 2010;303:841–8 [91]	AAA		✓					
ARRIVE Executive Committee, et al. Lancet 2018;392:1036–46 [5]	ARRIVE		✓		✓		✓	
Ikeda Y, et al. JAMA 2014;312:2510–20 [92]	JPPP		✓					
McNeil JJ, et al. N Engl J Med 2018;379:1509–18 [6]	ASPREE		✓	✓	✓		✓	
Ogawa H, et al. JAMA 2008;300:2134–41 [93]	JPAD	✓	✓			✓		
Miedema MD, et al. Circ Cardiovasc Qual Outcomes 2014;7:453–60 [94]	MESA		✓					
Ridker PM, et al. N Engl J Med 2005;352:1293–304 [95]	WHS^†^				✓			
Historical RCTs*								
Roncaglioni MC; Collaborative Group of the Primary Prevention Project. Lancet 2001;357:89–95 [96]	PPP^†^				✓			
Peto R, et al. Br Med J (Clin Res Ed) 1988;296:313–16 [97]	BMD^†^				✓			
Steering Committee of the Physicians’ Health Study Research Group. N Engl J Med 1989;321:129–35 [98]	PHS^†^			✓	✓			
The Medical Research Council's General Practice Research Framework. Lancet 1998;351:233–41 [99]	TPT^†^				✓			
Hansson L, et al. Lancet 1998;351:1755–62 [100]	HOT^†^				✓			
ETDRS Investigators. JAMA 1992;268:1292–300 [101]	EDTRS	✓				✓		
**Modeling studies**								
Dehmer SP, et al. Aspirin Use to Prevent Cardiovascular Disease and Colorectal Cancer: An Updated Decision Analysis for the US Preventive Services Task Force. 2022. AHRQ publication 21–05,283-EF-2 [102]				✓				
Dehmer SP, et al. JAMA. 2022;327:1598–1607 [103]				✓				

^⁎^ “Historical” and “contemporary” classification based on enrollment into study before 2000, according to Ridker 2018 [78]. ^†^Reference included as part of ATT 2009 meta-analysis [71].

ACC, American College of Cardiology; AHA, American Heart Association; DM, diabetes mellitus; ESC, European Society of Cardiology; RCT, randomized controlled trial; USPSTF, US Preventive Services Task Force.

Many important risk modifiers are not included in risk calculators, and there are major differences in the prevalence of risk factors across countries. Updated in 2019, the WHO CVD risk prediction charts reveal substantial variation in the estimated 10-year predicted risk for a given age and risk factor combination across the 21 global regions [36]. Similarly, findings of the Prospective Urban Rural Epidemiology (PURE) study reveal that the impact of some risk factors, such as low education, poor diet, and household air pollution, vary by the economic level of countries and were largest in middle- or low-income countries [104]. Although metabolic risk factors are the predominant individual-level risk factors for CVD [104], large ethnic differences in, for example, plasma lipid levels exist [105]. In addition, CVD risk in a population can be markedly affected by environmental changes [106]. Data from China show a complex geographical profile of CVD risk, with substantial variation and clustering of risk factors in some regions related to regional environmental and socioeconomic characteristics [107]. A comparison of CV risk factors in China and the USA showed that although China has a lower prevalence of CV risk factors, the burden is higher for hypertension, with poor detection and treatment, which may be responsible for China's high stroke prevalence [108]. Taken together, these and other data illustrate the difficulty in assessing the CV risk of an individual within an appropriate population risk level.

### 4.1 Frequency of low dose aspirin use in clinical practice

In the USA, data from the Behavioral Risk Factor Surveillance System found that the weighted prevalence of low dose aspirin use among adults without CVD was 18.6% (95% CI: 17.9%–19.3%) [109]. Use of low dose aspirin for primary prevention was more likely among males, patients aged ≥70 years, and patients who were overweight/obese despite low ASCVD risk burden, demonstrating suboptimal concordance between current recommendations and actual use patterns. Thus, there is evidence to suggest that low dose aspirin therapy is underused by those at high risk for CVD and overused by those at low risk for CVD [[110], [111], [112]], and, as highlighted by a recent transatlantic survey, most patients lack insight into their 10-year CV risk and do not know the risks, benefits and role of aspirin in CVD prevention [113]. In Europe, access to low dose aspirin for CVD prevention is not over the counter and requires input from a health care professional; in the USA, the low cost and over-the-counter availability of low dose aspirin facilitate self-medication [106].

Underuse of aspirin for the primary prevention of CVD has been estimated to occur in 31% to 84% of appropriate patients [114]. A recent survey reported that over half of patients with ≥10% CHD risk reported not using low dose aspirin despite treatment being indicated [110]. In the Multi-Ethnic Study of Atherosclerosis (MESA, years 2000–2002), regular low dose aspirin use was reported by 29% (CAC score 101–400) and 33% (CAC score >400) of individuals for whom this preventive therapy was considered appropriate according to AHA guidelines [115].

## 5 Bleeding risk mitigation strategies in China, the USA, South Korea, Italy, and Europe

A meta-analysis of trials evaluating aspirin for primary prevention of CVD, including the ASCEND, ASPREE, and ARRIVE studies, found that the use of low dose aspirin reduced CV events by 11% (hazard ratio [HR]: 0.89 [95% CI: 0.84–0.94]), with a number needed to treat to prevent 1 event of 241 [85]. However, it also increased major bleeding, such as serious gastrointestinal (GI) bleed, intracranial bleed or bleed needing hospitalization or transfusion, by 43% (HR: 1.43 [95% CI: 1.30–1.56]), with a number needed to harm of 210.

As upper GI complications, including ulcer and bleeding, are not uncommon during antiplatelet treatment, concomitant PPI treatment is often prescribed. However, the concomitant use of PPIs is not mentioned in the current ACC/AHA [16] or Italian [33] cardiovascular prevention guidelines. The US Preventive Services Task Force recommendation statement [70] notes that PPI use may decrease the likelihood of hospitalization from a major bleeding event. The Chinese consensus statement goes further and identifies the consideration of PPI use and eradication of *Helicobacter pylori*, which is a common infection in that global region, as 1 of the 4 measures that have to be in place before the initiation of low dose aspirin treatment [61]. The ESC/EASD guidelines on diabetes, prediabetes, and CVDs provide a grade IIa recommendation for PPIs to be considered to prevent GI bleeding when low dose aspirin is used [47]. In South Korea, the Korean College of Helicobacter and Upper Gastrointestinal Research recommends PPI use and *H. pylori* eradication for patients with a history of peptic ulcer or bleeding starting long-term low dose aspirin therapy for secondary but not primary prevention [116]. The Helicobacter Eradication Aspirin Trial is currently testing the hypothesis that *H. pylori* eradication in patients using low dose aspirin will halve the incidence of subsequent adjudicated peptic ulcer bleeding that results in hospitalization [117].

Notably, although gastroprotection using a concomitant PPI can reduce the risks of upper GI ulcers (odds ratio [OR]: 0.16; 95% CI: 0.12–0.23) and bleeding (OR: 0.27; 95% CI: 0.16–0.43) compared with other treatments or placebo in patients taking low dose aspirin [118], none of the randomized controlled trials used in the development of primary prevention guidelines uses a structured approach to gastroprotection; for example, only 14% of patients were receiving PPIs at the start of the ASCEND study [63]. Physician awareness of and adherence to recommendations to prescribe PPIs regularly to people taking low dose aspirin (in secondary prevention) is low [119,120]: one study reported that more than 50% of the patients with an increased GI risk were not treated sufficiently with a concomitant PPI, increasing the risk of GI side effects [119].

As the benefits of PPIs are not affected by concomitant low dose aspirin therapy, this suggests the potential for the use of PPIs in older patients, for whom low dose aspirin therapy is often contraindicated due to a high bleeding risk [121].

However, several potential adverse effects of concern have been associated with long-term PPI therapy, including increased risk of fractures, pneumonia, CKD, and dementia, and more research is needed to clarify the mechanisms and clinical relevance of these potential PPI-related effects [122]. Therefore the issue of gastroprotection with PPIs remains a matter of debate.

## 6 The risks vs. benefits of low dose aspirin

The decision to initiate low dose aspirin for primary prevention of CVD requires formal structured consideration of both absolute benefits and harms of treatment. ASCVD events and bleeding episodes may not, however, have equal effects on long-term health [123]. The net benefit of low dose aspirin increases as the equivalence of severity of bleeding harms and CV benefits is altered—for example if 1 major CVD event were considered to be equivalent to 2 major bleeds [123]. A personalized treatment decision thus needs to take into account the attitudes to risk and benefit, as well as the preferences and circumstances, of the individual patient [124]. Unlike many heart attacks and strokes, GI bleeding associated with low dose aspirin is usually an acute non-fatal event, commonly followed by complete recovery [125]. Elwood et al. (2016) proposed that a more appropriate evaluation of the risk–benefit balance would be based on fatal adverse events, rather than on the incidence of bleeding. The distinction between GI bleeding and fatal bleeding is not trivial: spontaneous GI bleeds carry a higher risk of death than GI bleeds attributable to aspirin [125]. A meta-analysis of 11 randomized trials concluded that the majority of the adverse events caused by aspirin are GI bleeds, and there was no definitive evidence that the overall frequency of fatal GI bleeds is increased by low dose aspirin; the substantive risk for prophylactic low dose aspirin was identified as cerebral hemorrhage, with an estimated risk of 1 death and 1 disabling stroke for every 1,000 people taking aspirin for 10 years [125].

## 7 Conclusions

Guidelines and prediction systems for the assessment of CV risk are important but, as reviewed here, differ widely in terms of the risk factors included in their respective prediction models, their output parameters, and the risk modifiers considered. There are also differences in the definitions of risk stratification, targets for major risk factors, and the use of CAC score to identify high-risk, asymptomatic patients. The risk-based approach to CVD prevention requires a personalized evaluation of the total CV risk and is dependent on clinician awareness of the relevant factors. In this respect, the use of a reliable, contemporary risk prediction chart or calculator appropriate to the relevant region, country, ethnicity, and culture, such as Globorisk, is essential as a starting point for further personalization of risk. Patients with no clinical manifestations of CVD, such as those with longstanding diabetes, early-onset diabetes, diabetes with target organ disease, or with advanced subclinical atherosclerosis, may be “CAD-equivalent” in terms of CV risk, but are not identified by assessment of traditional risk factors and the commonly used prediction models.

Recommendations regarding the use of low dose aspirin in patients at high risk of CVD vary between guidelines, and between the presence and absence of T2DM. In addition, the use of concomitant PPI treatment, which may reduce the GI bleeding risk and therefore allow more patients to be considered for low dose aspirin treatment, is variable. A more structured approach to gastroprotection should be used in patients being considered for primary prevention of CVD with low dose aspirin, although more research is needed on the long-term safety of concurrent PPI use.

Stronger primary care initiatives are needed to ensure that patients, particularly adults aged 40 years or older with one or more of the traditional risk factors and patients with T2DM, undergo regular assessment for ASCVD risk and that their use of primary prevention measures, including low dose aspirin therapy, is optimized. Patient education to better inform about the benefits and, where use is not medically indicated, risks of regular low dose aspirin is also needed.

A more unified, consistent, and global approach to primary prevention of CVD with appropriate identification and assessment of risk factors in individual patients could improve guideline applicability, patient understanding and acceptance of therapy, and outcomes. CVD prevention guidelines generally do not account for the extent to which risk factor control is influenced by lifestyle, patient compliance with pharmacological treatment, or socioeconomic factors. This is particularly relevant in low-income countries, where appropriate pharmacotherapy is less widely available. Because the relative CV risk reduction is a function of baseline risk, the relative contribution of an inexpensive drug such as low dose aspirin to risk reduction may be higher than currently appreciated based on clinical trial results, its use in higher income countries, or use without adequate assessment of high-risk asymptomatic individuals. Although the role of aspirin in the primary prevention of CVD is not as large as previously thought, with improved detection of subclinical atherosclerosis and more formal structured assessment of bleeding risk, there may still be a place for low dose aspirin, particularly in patients who need a more personalized treatment approach. (Fig. 1)

**Fig. 2:**
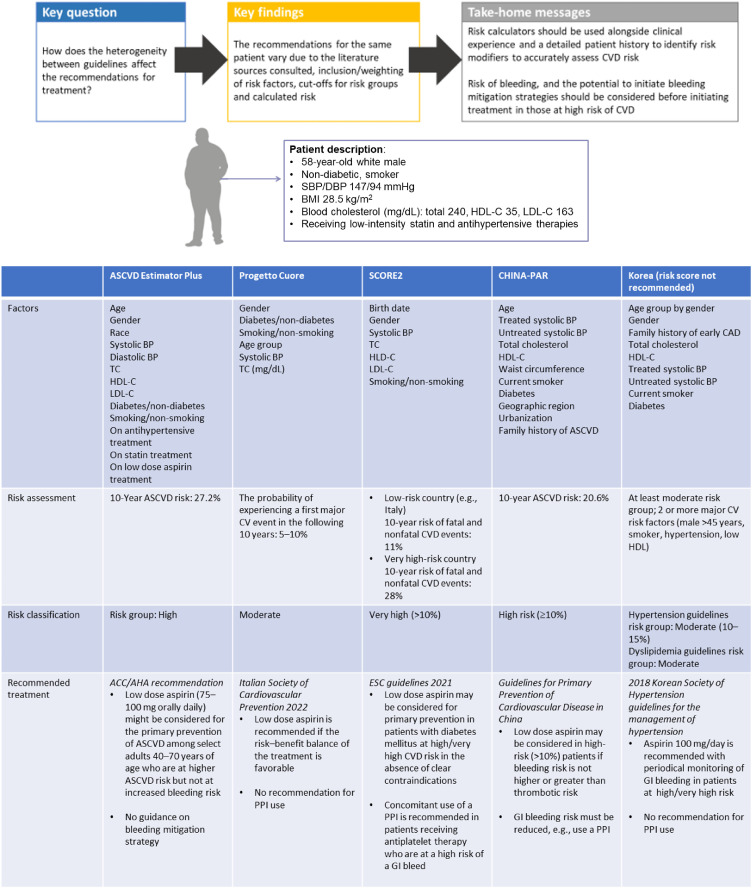
**Heterogeneity of recommendations for primary cardiovascular prevention across major clinical management guidelines.** This scenario depicts the recommendations from primary prevention guidelines for the USA, Italy, Europe, China and South Korea for the same hypothetical patient with several CVD risk factors. The recommendations are based upon the patient's high risk of a CV event and the guidance, where given, regarding the benefit:risk balance of low dose aspirin. ASCVD, atherosclerotic cardiovascular disease; BMI, body mass index; CAD, coronary artery disease; CVD, cardiovascular disease; DBP, diastolic blood pressure; GI, gastrointestinal; HDL-C, high-density lipoprotein cholesterol; LDL-C, low density lipoprotein cholesterol; PPI, proton pump inhibitor; SBP, systolic blood pressure; TC, total cholesterol.

## Funding

No funding was provided for the development of this manuscript.

## Disclosures

ZS, FS, MB and SHN are part of a Bayer AG global advisory board that convene to discuss risk assessment and preventive pharmacotherapy in primary and secondary prevention. MB declares an investigator-initiated grant funding from Bayer AG. LL declares employment by Bayer AG. The remaining authors declare that they have no other conflicts of interest.

All authors report writing assistance was provided by Highfield Communication Consultancy Ltd which was sponsored by Bayer.

## CRediT authorship contribution statement

**Xiao-Ying Li:** Conceptualization, Data curation, Formal analysis, Writing – original draft, Writing – review & editing. **Li Li:** Conceptualization, Data curation, Formal analysis, Writing – original draft, Writing – review & editing. **Sang-Hoon Na:** Conceptualization, Data curation, Formal analysis, Writing – original draft, Writing – review & editing. **Francesca Santilli:** Conceptualization, Data curation, Formal analysis, Writing – original draft, Writing – review & editing. **Zhongwei Shi:** Conceptualization, Data curation, Formal analysis, Writing – original draft, Writing – review & editing. **Michael Blaha:** Conceptualization, Data curation, Formal analysis, Writing – original draft, Writing – review & editing.

## Declaration of Competing Interest

The authors declare that they have no known competing financial interests or personal relationships that could have appeared to influence the work reported in this paper.
